# General Dental Practitioners’ Knowledge about the Emergency Management of Dental Trauma

**Published:** 2014-10-07

**Authors:** Najmeh Akhlaghi, Nosrat Nourbakhsh, Abbasali Khademi, Leila Karimi

**Affiliations:** a *Torabinejad Dental Research Center, Department of Pediatric Dentistry, Dental School, Isfahan University of Medical Sciences, Isfahan, Iran;*; b *Torabinejad Dental Research Center, Department of Endodontics, Dental School, Member of Iranian Academy of Medical Sciences, Isfahan University of Medical Sciences, Isfahan, Iran;*; c *Dental School, Isfahan University of Medical Sciences, Isfahan, Iran*

**Keywords:** Dental Emergencies, Dental Injuries, Dental Trauma, Emergency Treatment, General Dental Practitioner, Knowledge Assessment, Traumatic Dental Injuries

## Abstract

**Introduction:** The aim of this descriptive cross-sectional study was to assess the general dental practitioners (GDPs)’s knowledge regarding the emergency management of traumatic dental injuries (TDI) in Isfahan, Iran. **Methods and Materials:** In this study a two-part questionnaire consisting of 14 questions was distributed among 241 GDPs. Part 1 included seven questions focusing on personal and professional information and part 2 asked questions about seven given cases of dental traumas. One score was dedicated to each correct answer; the total score of 0 to 4 was considered as poor knowledge, while scores 5-8, 9-11 and 12-14 were assigned as moderate, good and excellent knowledge, respectively. The data were analyzed using the Student’s t-test and one-way ANOVA. Spearman’s and Pearson’s correlation coefficient were used to determine the associations between the emergency treatment knowledge and dentists’ professional information. **Results**: With regards to the level of GDP’s knowledge, the mean score was 7.61±2.68 suggesting a moderate score; a total of 177 (73.2%) of the dentists showed a moderate level of knowledge. A significant association was found between the frequency of dental trauma cases that were encountered and treated by GDPs in their daily practice (*P*=0.004, *r*=0.2). **Conclusion: **The overall knowledge of GDPs about the emergency management of TDI in the selected community was moderate.

## Introduction

Traumatic dental injuries (TDI) usually occur in the anterior segment and have a significant role in physical and psychological health [1]. Appropriate and immediate intervention would improve the prognosis and success rate of treatment in TDI. Thus, general dental practitioners (GDPs) must be adequately trained in this regard [2]. Different studies in Iran have shown that falling (56.5%), followed by sport and physical exertion (30.4%) [3] are the most common causes of TDI in 8-9 year-old primary school students; traumas mostly occur in 67.2% and 32.8% of boys and girls, respectively [4]. Dental injuries can be categorized into two major groups: hard tissue injuries including complicated and uncomplicated crown fractures, crown/root fractures and root fractures. The other group includes the injuries occurring in the PDL and alveolar supportive tissues, such as avulsion and luxation injuries.

Crown fracture of permanent upper central incisors and luxation in primary teeth are the most common injuries [5]. Management of TDI can be challenging for GDPs as it is sporadic and occurs at times when the dentists do not expect it and hence are not prepared for it [6]. In addition, dentists’ knowledge regarding the management of dentoalveolar traumas has an essential role in the prognosis of traumatized teeth because early intervention can enhance the regenerative capacity of traumatized teeth [7]. There are several worldwide studies evaluating the GDPs’ knowledge regarding the management and recognition of different types of dentoalveolar injuries; the majority of which have shown that dentists’ knowledge is inadequate and they cannot effectively manage such cases [2, 7-15]. GDPs’ knowledge regarding the diagnosis and treatment of different dentoalveolar injuries has been evaluated in different countries. South American GDPs’ knowledge in management of traumatized upper permanent teeth in children is reported to be moderate [12]. Kostopoulou and Duggal [13] suggested that GDPs’ knowledge was inadequate in the UK and Krastl *et al.* [14] confirmed that German dentists’ knowledge is not helpful in the management of traumatized teeth. Considering all the above mentioned explanations and emphasizing that GDPs’ knowledge about the management of traumatized teeth can dramatically improve the treatment prognosis and as there is no national published research in the literature in this regard, this study was undertaken to investigate the GDPs’ knowledge about the emergency management of traumatized teeth in Isfahan, Iran.

## Methods and Materials

This descriptive cross-sectional study was approved by Isfahan University of Medical Sciences and Research Center Ethics Committee (Grant no. 393006). A total of 241 GDPs (~10% of all GDPs in Isfahan) who attended a continuing 2-day dental education course at the Dental School, Isfahan University of Medical Sciences, participated in this study. The data was collected by means of a multiple-choice questionnaire including 14 questions in two parts. The volunteers filled the questionnaire and were assured that their personal data would not be reported. The questionnaires were handed in under the supervision of the examiner and were collected immediately after answering and matched with correct answers according to accepted current literature. Each questionnaire was divided into two parts: Part I consisted of seven questions about personal and professional information, including gender, age, university, frequency of patients with TDI they encountered in their daily practice (frequent, occasional and very rare), attendance at educational courses of TDI (yes or no), location of their present professional practice (personal office, state clinics, health centers, other), willingness to receive training in the management of TDI at undergraduate or postgraduate level or trauma fellowship courses and dentists’ self-assessment regarding their knowledge on the management of TDI (comprehensive, sufficient and fragmentary).

Part II contained 7 imaginary dental trauma cases to collect information about dentists’ knowledge in the management of dental traumas. These questions were about the management of a complicated crown fracture in a permanent tooth with open apex after 2 days, an uncomplicated crown fracture of a mature permanent tooth immediately after trauma, apical-third root fracture of a permanent tooth with mature apex immediately after trauma, an intruded permanent tooth with open apex immediately after trauma, an extruded permanent tooth with closed apex, an avulsed permanent tooth with closed apex with extraoral dry time longer than 60 min in a 12-year-old patient and an avulsed permanent tooth with closed apex in a child whose parents called the dental office to seek for advice at the exact moment.

One score was dedicated to each correct answer; as a result, while scores 0-4 were considered as poor knowledge, scores 5-8, 9-11 and 12-14 were taken as moderate, good and excellent knowledge, respectively.

Content validity of the questionnaire was analyzed before data collection. Questionnaires were reviewed by 4 pediatric dentists and 3 endodontists to judge if the questions can fulfill the aims of the present study. In order to calibrate the coordination of each question, each of the experts were asked to score each question with a high, moderate and low or uncertain degree of coordination from one to three, respectively. In addition, experts were asked to write any comments or suggestions they might have. Questions that had earned a score of 2 or 3 were corrected or removed according to the experts and the final questionnaire was again confirmed by experts. Finally, for the formal validation, the expert group and 10 GDPs assessed the questions.

Cronbach’s alpha was used to analyze the reliability of the questionnaire. Therefore, the questionnaire was completed by 15 GDPs with an interval of 2 weeks and 84.34% level of reliability was calculated, which is an indicator of reliability of the questionnaire.

***Statistical analysis***

The analyses were processed using the SPSS software (Version 20, Chicago, IL, USA). Descriptive statistics were used to analyze and present the data. The Student’s t-test and one-way ANOVA were used to compare the knowledge of participants about different aspects of traumatic events. Pearson’s correlation coefficient was used to test the relationship between knowledge scores and the age of the practitioners. Spearman’s correlation coefficient was used to test the relationship between knowledge scores and dentists’ self-assessment knowledge (*α*=0.05).

## Results

A total of 241 questionnaires (filled by 113 females and 128 males in the age range of 25-61 years) with 89.3% response rate were analyzed. The demographic data of the participants and the mean knowledge score for each item (part I) are summarized in Table 1. Participants’ knowledge about the clinical management of traumatic events in cases 1-7 (part II) are summarized in Table 2.

The total mean knowledge score was 7.61±2.68. A significant direct relationship was observed between the frequency of TDI that practitioners encounter in their daily practice and correct answers (*P*=0.004, *r*=0.2). Attendance at continuing educational courses of dental trauma had a significant effect on the knowledge score as well (*P*=0.03). There was a relatively positive significant relationship between GDPs’ self-assessment of their knowledge and their age (*P*=0.01, *r*=0.2). The relationship between self-assessment and frequency of TDI cases managed in their daily practice was significant (*P*=0.01, *r*=-0.2).

**Table 1:** The mean (SD) of knowledge score (K) of GDPs regarding traumatic dental injuries (TDI) and demographic distributions of GDPs (*n*=241)

**Variables**		***P*** **-value**	**K Mean (SD)**	**Frequency (%) **
**Gender**	**Female**	0.11 b	7.08 (2.6)	113 (46.8)
	**Male**		7.61 (2.37)	128 (53.2)
**Age**	**25-39**	0.58 b	7.28 (2.66)	157 (65)
	**40-61**		7.5 (2.16)	84 (35)
**Univ** **ersity **	**Isfahan**	0.18 b	7.38 (2.68)	178 (73.8)
	**Others**		7.53 (2.2)	63 (26.2)
**Frequency of patients with TDI in the practice**	**Frequent**	0.004^a^	7.42 (2.58)	48 (20)
	**Occasional**		7.47 (2.29)	86 (35.6)
	**Very rare**		7.06 (2.38)	107 (44.4)
**Attendance at educational courses about TDI **	**Yes **	0.03^a^	7.33 (2.64)	67 (27.8)
	**No**		7.46 (2.44)	174 (72.2)
**Location of the present professional practice**	**Private office**	0.33^b^	7.51 (2.35)	156 (64.8)
	**State clinics**		6.92 (1.76)	53 (22.0)
	**Health care centers**		7.57 (2.43)	15 (6.2)
	**Other**		11.37 (2.92)	17 (7)
**Willingness to receive training in relation to the management of TDI**	**Under-graduate courses **	0.31 b	7.19 (2.26)	204 (84.7)
	**Post-graduate courses**		7.33 (2.78)	18 (7.5)
	**Trauma fellowship courses **		10.5 (3.96)	19 (7.8)
**Dentists’ self-assessment regarding their knowledge about TDI**	**Comprehensive**	0.96 b	5.5 (1)	8 (3.3)
	**Sufficient**		9 (2.27)	74 (30.7)
	**Fragmentary**		7.2 (2.53)	159 (66)
**Knowledge**	**Poor (4 correct answers) **		7.61 (2.680)	14 (5.8)
	**Moderate (5-8 correct answers)**			177 (73.2)
	**Good (9-11 correct answers)**			42 (17.7)
	**Excellent (12-14 correct answers)**			8 (3.3)

a *: statically significant;*

b *: not statically significant*

## Discussion

This study assessed the knowledge of GDPs in diagnosis and management of TDI. According to the results of the present study, the practitioners’ knowledge regarding the management of complicated crown fracture, extrusion, avulsed primary teeth and critical time for replantation of avulsed permanent teeth, was acceptable. However, their knowledge about the treatment of uncomplicated crown fracture, root fracture, intrusion and avulsion of permanent teeth and splinting duration was not satisfactory.

The majority of participants declared that they rarely encountered traumatic events in their daily practice, which is consistent with the results of other studies indicating that TDI occur infrequently and at occasions when practitioners are not prepared for their appropriate management [6, 14].

Based on the approved guidelines on the management of complicated crown fractures in immature teeth, partial pulpotomy is the treatment of choice for large pulp exposures (>2 mm) or when the pulp has been exposed to the oral environment for more than 24 h [16]. The results of the present study suggested that dentists’ knowledge is acceptable regarding the treatment of this emergency. High success rates have been reported in preserving the pulp vitality by means of partial pulpotomy after complicated crown fractures in immature permanent teeth [16-18]. This is consistent with the current recommended guidelines of the International Association of Dental Trauma (IADT) [19]; however, the preferred treatment for a complicated crown fracture of mature teeth is endodontic treatment [19, 20]. Only half of the dentists believed that root fractures must be immediately repositioned and splinted for a period of 3 to 4 weeks. This is supported by the literature which indicates that the prognosis can be improved with rapid treatment and close adaptation of the root segments [21]. Cvek *et al.* [21] demonstrated that, obturating the coronal and apical fragments will result in failure and inter appointment calcium hydroxide dressing followed by root canal obturation confined to the coronal segment, appears to be the best treatment modality.

**Table 2:** The questioner focusing on the knowledge of GDPs (*n*=241) about traumatic dental injuries and frequency (%) of answers

**Questions**	**Answers**	**N (%)**
**Treatment of complicated crown fracture of immature teeth after 2 days**	Pulpectomy	45 (18.6)
	Partial pulpotomy (correct)	183 (76)
	One-visit RCT	7 (3)
	Did not answer	6 (2.4)
**Medicament of uncomplicated crown fracture of immature teeth**	Calcium hydroxide or mineral trioxide aggregate (MTA) (correct)	198 (82.1)
	Formalin	4 (1.6)
	Ferric sulfate	8 (3.4)
	Zonalin	18 (7.5)
	Gutta-percha	2 (0.8)
	Did not answer	11 (4.6)
**Immediate treatment of uncomplicated crown fracture **	Partial pulpotomy	8 (3.3)
	Glass ionomer (GI) or composite dressing (correct)	103 (42.7)
	2-week follow-up and then deciding for final restoration or RCT	122 (50.7)
	Did not answer	8 (3.3)
**Treatment of root fracture**	Immediate repositioning, splinting and follow-up (correct)	123 (51)
	Immediate repositioning, RCT, splinting	24 (10)
	Immediate repositioning, 2-week follow-up and then splinting if needed	90 (37.4)
	Did not answer	4 (1.6)
**Splint duration in root fracture**	1-2 weeks	101 (41.9)
	More than 4 months	6 (2.5)
	3-4 weeks (correct)	120 (49.8)
	Did not answer	14 (5.8)
**Time for endodontic treatment in root fracture?**	At least 1-year follow-up and RCT of coronal segment if necrosis occurred (correct)	96 (39.8)
	Extirpation of pulp 1 week later and RCT of the coronal segment	80 (33.1)
	Extirpation of pulp at the first visit, use of calcium hydroxide for 1 week and then RCT of both segments	53 (22.0)
	Did not answer	12 (5.1)
**Immediate treatment in intrusion**	Immediate orthodontic repositioning	61 (25.3)
	Allow for spontaneous eruption of tooth and if no movement occurs in 3 weeks, rapid orthodontic extrusion (correct)	131 (54.4)
	Allow for spontaneous eruption of tooth and if no movement occurs in 3 weeks, surgical extrusion by forceps	32 (13.3)
	Did not answer	17 (7)
**Treatment of extrusion**	Immediate repositioning and splinting (correct)	198 (82.2)
	Grinding teeth off from occlusion and splinting	20 (8.3)
	Allow for spontaneous repositioning	16 (6.7)
	Did not answer	7 (2.8)
**Treatment of avulsed mature teeth with >1 h dry-time **	Removing necrotic tissue with gauze, RCT, removing the coagulum from socket with saline, immersing in 25% sodium fluoride, replanting and splinting, antibiotic therapy (correct)	118 (49)
	Removing necrotic tissue with gauze, replanting and splinting, antibiotic therapy	31 (12.9)
	Immersing in 25% sodium fluoride, removing necrotic tissue, RCT, replanting and splinting, antibiotic therapy	90 (37.3)
	Did not answer	2 (0.8)
**Instructions in avulsed mature teeth at the site of accident**	Replantation of tooth immediately, and if the replantation procedure cannot be performed at this time, the tooth can be stored in saliva of patient and to be referred to the dental office immediately (correct)	163 (67.8)
	Store the tooth in saline solution and refer to the dental office immediately	40 (16.9)
	Store the tooth in milk and refer to the dental office immediately	26 (10.2)
	Did not answer	12 (5.1)
**Patient came to the office. What is the next step?**	Replantation if not performed before, splinting, antibiotic therapy (correct)	116 (48.2)
	Thermal test, endodontic treatment, radiographic examination, splinting, antibiotic therapy	85 (35.2)
	Did not answer	40 (16.6)
**Splint duration of avulsed teeth which were immediately replanted**	7-10 days (correct)	163 (67.6)
	2 months	31 (12.9)
	4 months	32 (13.2)
	Did not answer	15 (6.2)
**When was endodontic treatment carried out?**	At emergency first visit	33 (13.7)
	7-10 days after replantation and before removal of splint (correct)	132 (54.8)
	After removal of splint until necrosis was found	53 (22.0)
	Did not answer	23 (9.5)
**Differences in the management of avulsed primary tooth in a 4-year-old child**	Yes(correct)	202 (83.8)
	No	27 (11.2)
	Did not answer	12 (5)

Nearly half of the participants would wait for spontaneous re-eruption of intruded teeth with incomplete root formation. Andreasen *et al.* [22] reported that active repositioning of the immature teeth with either orthodontic or surgical forces imposes devastating effects on the healing capability of an intruded tooth. However, the long-term outcome of the surgical exposure and endodontic treatment followed by orthodontic repositioning in a case of sever intrusive luxation was satisfactory [23]. In case of extrusion of mature permanent tooth, the majority of GDPs would reposition it immediately and splint the tooth as it has been declared to be the method of choice for extruded teeth [1]. The stage of root development at the time of injury, the duration of the extra-alveolar time and the storage medium have been shown to be the most important factors for long-term success of a replanted avulsed tooth [24, 25]. A large proportion of dentists showed inadequate knowledge regarding the treatment of avulsed teeth with complete root formation in a growing patient with more than 60 min of extraoral time. In trauma management guidelines of IADT, the treatment of choice for mature avulsed teeth with more than 60 min of extraoral time is removing the necrotic tissue with a piece of gauze, root canal therapy, removing the coagulum from the socket with saline, immersion in 25% sodium fluoride, replanting, splinting and antibiotic therapy [25].

The most critical factor in the treatment of some dental injuries is the time management which can affect the prognosis of traumatic teeth [26]. The majority of dentists correctly agreed to replant the avulsed tooth at the accident site rather than in the dental office. However, as the incorrect replantation of the tooth by non-trained people can jeopardize the success rate of replanted tooth, it would be rational if patients seek immediate professional help at the site of accident. Essentially, the shorter the time lapse between avulsion and replantation, the lower the risk of replacement root resorption and external inflammatory root resorption [26, 27]. In addition, the majority of GDPs in this study would splint avulsed teeth for 7 to 10 days (Table 2). This rate is shown to be higher compared to previous studies as they reported only 10-30% of clinicians would splint the tooth for that duration [9, 13]. Nearly half of the participants correctly declared that endodontic treatment within 7 to 14 days after replantation of an avulsed tooth with complete root formation, that was replanted within less than 1 h, is the best treatment approach and this method is consistent with the published guidelines [25, 26, 27]. The majority of GDPs reported that they would not replant an avulsed deciduous tooth, which is consistent with the current guidelines and recommendations of the IADT [25].

The results of the present study showed that the majority of professionals labeled their knowledge as fragmentary, which is consistent with two other published studies [14, 28]. However, moderate overall level of knowledge was demonstrated by evaluating GDPs. Therefore, comprehensive strategies seem mandatory to keep them up-to-date and improve their knowledge about TDI [11, 14]. Different approved guidelines can be developed and distributed in the form of brochures and posters to emergency and dental clinics to enhance clinicians’ and people’s knowledge [28]. In addition, specialized centers for TDI can be established in cities with some trained staff to offer 24-h services, especially for complicated cases. These centers must be mutually connected to GDPs of the city and they should be able to consult their cases and the patients could also be referred to dentists to follow the treatment process. As early intervention in the management of TDI can significantly change the outcomes of treatment, all the clinicians must be sufficiently trained to efficiently manage the traumatic events. Therefore, further studies on the effect of trauma-management courses on clinicians’ knowledge are suggested so that such courses can be extended for all the GDPs.

## Conclusion

This study demonstrated that general dentists in Isfahan had a moderate level of knowledge about management of traumatic dental injuries. Practical skills, experience and self esteem are effective on dentists’ knowledge.
